# ‘it was that … specialist … that finally listened to us … that's probably a weird answer to what you were expecting’: Clinician and carer perspectives on brilliant feeding care

**DOI:** 10.1111/hex.13683

**Published:** 2022-12-08

**Authors:** Ann Dadich, Simone Kaplun, Cathy Kaplun, Nick Hopwood, Christopher Elliot

**Affiliations:** ^1^ School of Business Western Sydney University Parramatta NSW Australia; ^2^ Transforming early Education and Child Health (TeEACH) Strategic Research Initiative Western Sydney University Westmead New South Wales Australia; ^3^ Faculty of Arts and Social Science University of Technology Sydney Broadway New South Wales Australia; ^4^ Department of Paediatrics St George Hospital Kogarah New South Wales Australia

**Keywords:** brilliant care, child health, feeding difficulties, tube‐feeding

## Abstract

**Introduction:**

To extend research on positive aspects of health care, this article focusses on health care for children who tube‐feed—this is because knowledge about tube‐feeding for children is limited and fragmented. This is achieved by consulting with clinicians and carers who supported children who tube‐feed to clarify their understandings of and experiences with brilliant feeding care.

**Methods:**

Nine clinicians and nine carers who supported children who tube‐fed were interviewed. The interview transcripts were analysed thematically.

**Results:**

Findings highlighted several features of brilliant feeding care—namely: practices that go above and beyond; attentiveness; empowerment; being ‘on the same page’; hopefulness and normalcy.

**Conclusions:**

These findings show that seemingly trivial or small acts of care can make a significant meaningful difference to carers of children who tube‐feed. Such accounts elucidate brilliant care as grounded in feasible, everyday actions, within clinicians' reach. The implications associated with these findings are threefold. First, the findings highlight the need for clinicians to listen, be attuned and committed to the well‐being of children who tube‐feed and their carers, share decision‐making, source resources, and instil hope. Second, the findings suggest that carers should seek out and acknowledge clinicians who listen, involve them in decision‐making processes, and continue to source the resources required to optimize child and carer well‐being. Third, the findings point to the need for research to clarify the models of care that foster brilliant feeding care, and the conditions required to introduce and sustain these models.

**Patient or Public Contribution:**

All of the carers and clinicians who contributed to this study were invited to participate in a workshop to discuss, critique, and sense‐check the findings. Three carers and one clinician accepted this invitation. Collectively, they indicated that the findings resonated with them, and they agreed with the themes, which they indicated were well‐substantiated by the data.

## 1 INTRODUCTION

Many children, worldwide, require a tube to maintain adequate nutrition, orally.^1^ Paediatric feeding disorder requiring tube‐feeding (PFD‐T)^2^ might involve the following: a nasogastric tube, which is inserted into the nose and through to the stomach; an orogastric tube, which is inserted into the mouth and through to the stomach; or a percutaneous endoscopic gastrostomy tube, which is surgically inserted into the stomach. Although it is difficult to estimate the prevalence of PFD‐T, it is said to be between 1 and 4 children per 100,000.^3^ However, this rate can be as high as 83–92 per 100,000, if not more.^4^

Despite the prevalence of PFD‐T, knowledge about it is far from complete. This is largely due to two reasons. First, there are over 350 health conditions that can warrant tube‐feeding.^5^ These include (but are not limited to) cerebral palsy, neurodevelopmental disabilities, cleft palate, cystic fibrosis, prematurity, recovery postsurgery, and ill health.^6^, ^7^, ^8^ As such, ‘There are multiple, complex pathways to paediatric tube‐feeding’.^2^ ^,p. 1^

Second, different clinicians affiliated with different specialities manage tube‐feeding, conceptualizing it differently.^9^, ^10^ With few exceptions,^11^, ^12^ research on PFD‐T tends to focus on particular health conditions.^13^, ^14^, ^15^, ^16^, ^17^, ^18^ Consequently, knowledge about PFD‐T remains fragmented.

Regardless of why a child requires tube‐feeding or the specialities involved in their care, PFD‐T can have personal, social, and economic implications. It can isolate the child and their family from social interactions; compromise the child's well‐being; generate carer anxiety, family strain, and relationship issues; as well as warrant greater access to (mental) health services, adding to rising healthcare costs.^4^, ^13^, ^19^, ^20^, ^21^, ^22^, ^23^ Furthermore, feeding difficulties and the management of tube‐feeding among children are not always readily ‘fixed’ by health services. This is due to myriad reasons, including poor clinician recognition of carer concerns.^24^ For instance, researchers have noted that ‘Most of the primary caregivers… found it difficult to coordinate care and obtain support when needed’,^25^ ^,p.25^ and ‘parents could benefit not only from sensitive and respectful collaboration but also from anticipatory guidance’.^26^ ^,p.212^

This literature, which primarily awards attention to problems and issues, depicts a somewhat bleak portrayal of feeding difficulties and tube‐feeding. While it is important to identify problems and issues, a preoccupation with all that is wrong with healthcare can itself be a problem. For instance, for patients and carers, a continued focus on that which is negative can silence their positive experiences with health issues and/or health services—and there are many instances of these^27^; it can also diminish help‐seeking behaviours and subsequent access to timely care.^28^ For clinicians and service managers, this preoccupation risks unfairly stereotyping them as part of a systemic problem^29^, ^30^—furthermore, it can diminish learning opportunities and innovation.^31^ And for policymakers, it might continue to direct their attention (and public funds) to ineffective and/or inefficient healthcare practices—this is because, rather than problematize beliefs and assumptions, the identification of problems and issues is largely based on prevailing beliefs and assumptions, leaving little opportunity for innovation.^32^

Building on the literature that visibilises ‘that which is positive, flourishing, and life‐giving in [healthcare] organisations’,^33^ ^,p.731^ and redresses the scholarly preoccupation with the problems and issues in feeding care, this article purposely considers what constitutes brilliant feeding care.^34^, ^35^ This is achieved by consulting with clinicians and carers who support children who tube‐feed. The article commences with a brief overview of brilliant care. After describing the study focus and the research method, the findings on what constitutes brilliant feeding care are presented. The article concludes by explicating the implications associated with these findings for scholars, clinicians, and carers.

### 1.1 Brilliant care

Brilliant care can be conceptualized in ways that are not tied to specific health outcomes. It is a relational experience that exceeds expectations, bringing joy and delight to those who experience or witness it.^36^ Brilliant care can be unconventional and serendipitous, and does not necessarily represent business as usual within a service or a sector. Furthermore, brilliant care is interpersonal, uplifting, inspiring, and/or energizing.^37^

Aspiring for brilliance in care goes deeper than meeting or exceeding performance indicators. One aspect of this involves the recognized benefits of positive emotions in diverse contexts, including healthcare. Fredrickson's broaden‐and‐build theory helps to understand this important feature of brilliance—‘Positive emotions… *broaden* people's momentary thought‐action repertoires and *build* their enduring personal resources’ (p. 147, original italics).^38^ The experience of healthcare can benefit from upward spirals as positive emotions and the expanded thinking they promote become mutually reinforcing.^39^

A second important aspect of brilliance concerns an ethic of care. An ethic of care awards primacy to connections.^40^ It recognizes the importance of ‘trust and responsibility, protection of individuality, the context in which the relationship takes place, and the quality of the relationship’.^41^ ^,p.3^ Furthermore, it recognizes listening as a way to fortify trust, strengthen relationships, and diversify voices.

Of particular relevance to brilliant care is the resistance that an ethic of care epitomizes—it counters assumptions and norms that sustain injustice.^42^ It recognizes a need to ‘negotiate relations between self and other in ways that resist the hierarchies that maintain existing relations of power’.^43^ ^,p.13^ Correspondingly, brilliant care defies what might be expected to foster connections that enable individuals or collectives to flourish.^34^ With this theoretical backdrop, this article considers what constitutes brilliant feeding care according to clinicians and carers who supported children who tube‐feed.

## 2 METHOD

Following clearance from the relevant human research ethics committee (approval number: H13794), clinicians and carers who supported children who tube‐fed were invited to participate in a semi‐structured interview. Clinicians were primarily recruited via purposeful sampling. Clinicians aged 18 years or older, who resided in Australia, and had spent most of their working week engaged in feeding care for children aged under 18 years, were invited to participate in this study via email. Carers were recruited via social media platforms (e.g., Facebook, Twitter) and relevant webpages. Carers were invited to contact the researchers to participate in this study if they were aged 18 years or older; resided in Australia; and cared for a child aged under 18 years who required tube‐feeding within the last 5 years (to optimize the currency of the findings). Participant recruitment of both cohorts continued until data saturation.^44^ Specifically, data analysis occurred in tandem with data collection and when ‘no new information, codes or themes … [were] yielded from the data’ (p. 202), recruitment efforts ceased.

The researchers devised two interview schedules, one for clinicians and one for carers (see Appendix 1). The schedule for the clinicians pertained to the following: how they became interested in feeding difficulties and/or tube‐feeding; what they have found useful when supporting children who tube‐feed and/or their carers; their understandings of and experiences with brilliant feeding care; and what they wish they would have known about feeding care, earlier in life. The schedule for the carers pertained to: the lived experiences of tube‐feeding; what helped or hindered feeding care; the priorities and considerations that mattered to them; their understandings of and experiences with brilliant feeding care; and what they wish they had known about feeding care, earlier in life. Given the article's focus, only findings pertaining to brilliant feeding care are presented. To ensure the schedules were fit‐for‐purpose, this study and the schedules were discussed with members of the SUCCEED Child Feeding Alliance. The SUCCEED Child Feeding Alliance represents a unique collaboration between health professionals, academics, artists, and families who are passionate about supporting children with feeding difficulties and their families. Alliance members were invited to consider and critique the study design and inform the development of the schedules.

Following informed, written consent, nine clinician and nine carer interviews were conducted via web conferences for approximately 1 h (see Table 1). The interviews were digitally recorded and transcribed for thematic analysis.^45^ One researcher (re)listened to the recordings as well as (re)read and reviewed the transcripts to ascertain patterns within the dataset. They also constructed broad (or higher‐order) themes that reflected participant experiences and perceptions. To clarify their understandings of and experiences with brilliant feeding care, particular attention was awarded to experiences that brought joy and delight; ‘*broaden[ed]* people's momentary thought‐action repertoires and *buil[t]* their enduring personal resources’^38^ ^,p.147^ (original italics); bolstered connections, and exceeded expectation by defying norms.^46^ This process was aided by NVivo 12—computer‐assisted qualitative data analysis software.^47^ To optimize the veracity of the analysis, two other researchers analysed half of the transcripts each. The three researchers conferred about their respective themes and reconciled differences.

**Table 1:** Participant demographic details and attributes

Cohort	Characteristic	*n* (%)
Clinicians (*n* = 9)	Age (years)	
	20–29	2
	30–39	3
	40–49	3
	50–59	1
	Gender (female)	8 (88.9)
	Geographical location	
	Queensland	9 (100.0)
	Discipline	
	Dietetics	7 (77.8)
	Speech pathology	2 (22.2)
	Experience in child health care (years)	
	1–5	2
	6–10	3
	11–15	1
	16–20	2
	Over 30	1
	Employed in a tertiary health service	3
Carers (*n* = 9)	Age (years)	
	30–39	5
	40–49	2
	50–59	2
	Gender (female)	8 (88.9)
	Geographical location	
	New South Wales	2 (22.2)
	Victoria	2 (22.2)
	Queensland	2 (22.2)
	Unspecified	3 (33.3)
	Employment status	
	Full‐time employed	3 (33.3)
	Part‐time employed	3 (33.3)
	Unemployed	2 (22.2)
	Retired	1 (11.1)
	Experience in supporting children with feeding disorders (years)	
	0.5–2	4
	3–3.5	4
	15	1
	Age of child with a feeding disorder (years)	
	1–2	4
	3–3.5	4
	15	1
	Gender of child with a feeding disorder (male)	6 (66.7)

## 3 RESULTS

Findings from the 18 interviews highlighted six features of brilliant feeding care. Each is addressed in turn.

### 3.1 Going above and beyond

Brilliance was aptly demonstrated when others went above and beyond their substantive role to support a child who tube‐fed or their carer. Participants described individuals in their lives who ventured outside their remit to perform acts of care. Sometimes these acts were considerable—they required significant time and effort or placed the individual in a potentially precarious situation:

[My son] had a really… bad turn… his heartbeat and his breathing just almost stopped… the nurse… called a [medical emergency team]… It wasn't until about two in the morning that I was just standing there watching them… and… his paediatrician just appeared at my shoulder… I was like, ‘What are you doing here? You're not oncall’. She said… ‘I just came in to make sure everything was okay’… She was there all night until eight in the morning and then did a full day at work. She just came in to make sure that [my son]… was okay. (*carer 14*)

Equally important were small acts of care—deeds that perhaps did not require the individual to invest considerable time and effort or place themselves at risk, but nevertheless made a sizeable impression on others:

there was just these really… small little details that she gave us that made a big difference to make sure that we were… doing the right thing for [our son]. (*carer 15*)

The significant and relatively minor acts of care shared two features. First, the instances typically occurred during times of heightened adversity. For instance, they occurred when a carer experienced considerable strain, distress, or anxiety. During these moments, brilliant care was a helpful antidote:

the one thing that's standing out for me is the parent who said… ‘You're the first person who's listened to me and believed that this is a real thing and a real issue, and… told me that it's not my fault or that I'm not being paranoid’… I think listening and really unpacking that with them can have such a big impact. (*clinician 11*)

Second, the acts of brilliant care exceeded expectations. In contrast with the healthcare they were used to, which was often rigid, the carers were moved by displays of care. They were inspired and encouraged by those who acted compassionately, transcending the pedestrian pattern of healthcare that they and their child typically received:

our first paediatrician… told us … [my son]… had silent reflux—‘Go home and take this… He will be fine’… we went back… two weeks later and I was like, ‘Look, it's getting worse’. So, then he tried us on this… formula. Again, it almost made him worse… then I attempted to see a third paediatrician. They told me the same thing… I had an appointment with our baby health nurse… we weighed him and… she had this look on her face and I said to her… ‘What?’… she just said, ‘I'm sorry… As a baby health nurse, we can't give recommendations and advice’… I said to her… ‘what's the problem right now?’ And she said, ‘He has just tipped under the third percentile’… I just said… ‘If I said to you, I'm going to get a third opinion, would you say that I'm doing the right thing?’ And she said, ‘Yes’. I said… ‘If I said to you that I was going to attempt to… [see Dr A or Dr. B]… what would you say?’… she said, ‘You are a fantastic mum… you will know what to do’… I rang [both doctors]… no one picked up, so I left a message… I got a phone call back… from [Dr A's] … rooms… the lady… at the front desk… she said, ‘Alright, now just hold on a moment. Just calm down… tell me what's happening’… I just said, ‘I need help… I need to save my baby’… she said, ‘Look, [Dr A]… is not in today, but I will call him and I will get him to see you on Monday’… I got a call back from [her] … and she said… ‘I've just spoken to [Dr A] … and he said if you can be in his room on that Saturday morning at nine o'clock, he will see you’… so we went… and … said to him, ‘Look … we have been through the ringer… no one is helping us. If you can't help us today, our car is actually packed and we are going to [the hospital]’… he… said, ‘…you are not going anywhere; I will be escorting you to hospital’. And so, he actually did and from that point onwards we didn't leave hospital for the ten weeks… we are so incredibly grateful for him and our baby nurse… she subtly gave me the hints of what would be best. And if she ever got into trouble for any of that, I would back her a 1000% because without her and [Dr A]… we actually don't know where we would have been. (*carer 15*)

This account demonstrates the complementary roles of different forms of brilliant care. Mindful of what she was (not) permitted to do, the nurse used praise to gently nudge the carer to source alternative medical advice; while the doctor discernibly and proactively strived to attend to the carer's concerns.

### 3.2 Attentiveness

Several carers described the positive impact of clinician attentiveness. Attentiveness was important because it indicated that the carer and child well being mattered. Rather than prioritize their own interests, like managing limited time or assuming what others needed, the clinicians were thoughtful and they considered what the carer and child might need:

it was that renal specialist in terms of the feeding that finally listened to us… that's probably a weird answer to what you were expecting. (*carer 1*)

Attentiveness was demonstrated directly and indirectly. The former included the following: the respectful questions that others asked and how they deferentially asked them; how they fulfilled promises, such as sourcing supplies or clinical expertise; and unsolicited offers of support. Indirect attentiveness included others' observations—how they noticed the signs that a child's health might be compromised, or that a carer might be struggling with the complexities of feeding care.

Demonstrations of attentiveness were deemed brilliant for two reasons. First, they exceeded expectations. They did not reflect the norms of mediocre or dismissive healthcare that carers were accustomed to. Instead, others' thoughtfulness was serendipitous, positively deviating from what the carers expected:

just the fact that the doctor… was actually asking what I thought. ‘What do you think… would help? What about this? Has he tried that?’… they were very open; whereas, I find… with different doctors… it's very much, ‘I'm a doctor. I know what… is needed’… not really listening to what your experiences are, what you know that child needs. (*carer 2*)

Second, because managing a feeding disorder can be overwhelming and exhausting, carers were not always able to recognize or articulate their needs. Carers sometimes needed a carer—someone to look out for them, respectfully identify what might be helpful, and support them:

I can just see that parent has had zero sleep, [so I] rework… the plans to make it work… maybe we need to change the overnight [feeds]… to continuous… things like that really help. Sometimes the families aren't in a space to articulate that goal at that particular moment because they're so sleep deprived. But they come back at the next review, and they are glad that we made the change. (*clinician 5*)

### 3.3 Empowerment

According to the participants, brilliant feeding care was demonstrated by empowerment—when they or others experienced improved confidence and were better able to exercise agency. Unfamiliar with and uncertain about feeding difficulties or feeding care, clinicians and carers often struggled to know what to do and how to do it. The associated insecurity and anxiety were sometimes exacerbated by an awareness that, just as a child's failure to thrive can be distressing and dangerous for carers and their child, so too can tube‐feeding. Tube (re)insertion can be distressing and uncomfortable for the child—it can also be dangerous if performed incorrectly. A clinician's or carer's feelings of helplessness and hopelessness often subsided when they were encouraged and supported to take greater control over an uncertain or anxiety‐provoking situation. Sometimes, this made a world of difference:

what made it really helpful or empowering, was the fact that it was so much about learning to trust your child… ultimately trying to… empower… families and… children… just in terms of knowledge, just in terms of understanding the experiences you're going through, in terms of helping you to find your own way forward with things, that was a really brilliant experience. (*carer 8*)

Empowerment was typically facilitated by clinicians and other carers. Participants described how these individuals reassuringly shared advice, enabling them to manage difficult situations, feel prepared, and gain a greater sense of control:

We… had a buddy system… particularly for those littlies that were going through tube‐wean. So, successful tube‐weaners would then buddy with families… prior to achieve wean, so they could provide some additional support. I think that worked really well because… hearing it from clinicians is quite different to hearing it from a parent that's had a lived experience. (*clinician 1*)

In the context of empowerment, although the advice was important, so too was the way it was offered. Given that health education was typically offered prescriptively, encouragement and reassurances were welcome juxtapositions:

The [percutaneous endoscopic gastrostomy or] PEG team at the hospital… were awesome… they teach you how to put the PEG in and out by yourself. Just teach you everything about it and make you feel comfortable with it… they… say, ‘You're doing a good job, you're doing awesome’… before that, no one ever said stuff like that, ever. (*carer 11*)

### 3.4 ‘We're all on the same page’

Brilliant feeding care involved having a shared understanding with others of what mattered and how to realize aspirations, particularly with those who contributed to the child's care. This was important given that evidence‐based child healthcare requires a multidisciplinary approach.^48^ As such, several clinicians who represent different disciplines are typically involved in the care of a child who is tube‐fed. Despite the potential value of complementary areas of expertise, some participants noted how overwhelming and confusing multidisciplinary care can be—this was largely because different clinicians often espoused different opinions (in different ways) on how to best manage a feeding difficulty. However, when clinicians and carers worked as a team towards shared goals, brilliant care was experienced:

we're all working towards the same goal… we're all on the same page and that's the positive that I take out of all the back and forth with everybody else. (*carer 4*)

Being ‘on the same page’ was considered brilliant because it surpassed the confusion and inefficiency that many clinicians and carers were used to. When clinicians or carers felt understood, they did not feel obligated to explicate their concerns or experiences at length or repeatedly. The discussion was relatively easier because there was an unstated recognition of what was typically a complex situation, and there was sympathy for those attempting to manage such complexity:

there's no chopping and changing with that department. It still is the same lady… when there's chopping and changing and it's a different person every week, you feel like you're starting from scratch every week and you've got to tell them his… life story to get to the point, every single time… it's always been the same person. That makes a massive difference because she knows his needs. (*carer 11*)

### 3.5 Hopefulness

Participants indicated that brilliant feeding care was demonstrated when they were inspired or offered hope. Depleted by the challenges of caring for a child with a complex health condition, their confidence and their aspirational outlook on life often waned. Yet, this situation and their outlook could be considerably altered when they experienced a semblance of optimism. For instance, when carers felt disheartened, clinicians made a positive impact by working with the carer and child towards feasible goals. Through reassurance and goal achievement, carers felt better equipped to manage their difficult circumstances:

I recently had a little four‐month‐old bub… she couldn't feed because of her reflux… we did really… well with her because, at the beginning of inserting the tube, we made… three‐month goals that… helped guide what we do… that has gone… really well, because the goals that we made were really appropriate for the baby and the family. (*clinician 6*)

According to the participants, the goals need not be feeding‐related, but simply a small step that culminated with positive change. This was important because positivity begot positivity^38^—a positive change, even if small, whet a carer's appetite for more change:

I like to think of us [clinicians] as their cheer squad to celebrate those wins with them. (*clinician 9*)

### 3.6 Normalcy

Experiences that exceeded expectations often promoted normalcy. According to the participants, managing a feeding difficulty disrupted the lives that carers had expected for themselves and their children, and sometimes created chaos. The chaos was inflamed by the anxiety and confusion that carers can experience when their child has a complex health condition. And when they felt out of their depth, acts of care that offered a sense of manageability made a considerable difference:

when we'd gone to get his [gastrostomy] button changed with the public system, there was a nurse there and she was really, really good… I was really panicking… and thought it was going to be horrific… She just made everything seem so normal… she was like, ‘I'll just take this off and clean it and do that and do that’, and we were like, ‘Oh, okay, it's quite easy’… It was really, really good. (*carer 12*)

The significance of normalcy was also demonstrated beyond the confines of a health service. The carers and their children had myriad other relationships, be they with teachers, family members, friends, or community members. Participants noted that their expectations were exceeded when carers and their children felt normal and not shunned. This was important because they often felt stigmatized by others who did not understand feeding difficulties or why tube‐feeding was warranted. In contrast to such marginalization, opportunities to feel accepted and part of the collective brought joy. When carers and their children felt welcomed, their extraordinary feeding practices felt somewhat ordinary:

I saw a brilliant school that integrated all the tube kids into the canteen and all the kids had a menu, the same as everybody else. They knew what was going down their tubes… They could choose what they wanted, and they were part of the mealtime. (*clinician 4*)

## 4 DISCUSSION

This article clarified clinician and carer perspectives on what constitutes brilliant feeding care—care that exceeds expectations, fostering positive emotion and connections. Participants suggested that brilliant feeding care is bolstered by the following: practices that go above and beyond; attentiveness; empowerment; being ‘on the same page’; hopefulness, and normalcy. These ingredients often made a world of difference to carers and their child, particularly during times of heightened adversity.

These themes captured the importance of practices that went beyond the oft‐cited delivery of pedestrian or confusing care^21^ to provide an unexpected level of support in a sensitive and respectful way.^25^ Whether this involved taking extra time to listen to and understand carer concerns,^8^, ^24^ offering a kind word or gesture, or sourcing additional support, these unexpected practices demonstrated greater empathy and concern for the child and their carer. In essence, they charged care with brilliance.

Carers who experienced care that exceeded expectations were better able to manage the challenges of tube‐feeding. This speaks to an upward spiral,^39^ whereby positivity begot positivity—specifically, ‘the psychological state influenced the ability to cope with challenges, and challenges impacted the psychological state’.^21^ ^,p.8^ By stepping in and perhaps stepping up at these key times, those who facilitated brilliant feeding care influenced the child and carer's experiences.

As carer confidence grew with tube‐feeding, normalization became an aspiration for many. Normalcy was experienced when they felt: supported to manage their child's feeding difficulty; and accepted, particularly in public spaces. These experiences often incited joy. However, for this to occur, the carer required an understanding and knowledge of tube‐feeding. Care could then be integrated into a daily routine, with the hope of eventually forming a ‘new’ normal. Towards this aim, carers often sought the support of individuals who understood and supported their goals for their child.

The participants cited some of the barriers associated with fragmented care, with some recognizing brilliance in multidisciplinary care in which everyone was ‘on the same page’. This reflects research on the value of multidisciplinary child healthcare.^48^ Working as a multidisciplinary team also benefitted clinicians who appreciated the reduced burden of care when managing complex health issues. From the carers' perspective, this meant a consistent message from everyone involved in their child's care, reducing the confusion associated with receiving conflicting advice from disparate clinical voices.

Although this study focussed on brilliant feeding care, identifying it often required the participants to recount substandard care. Perhaps necessarily, they narrated the ordinary to personify the extraordinary. This was particularly the case when participants spoke of empowerment. For clinicians, rather than retain control over care, empowerment often involves encouraging others to exercise agency and support each other. For carers, a clinician's brilliant practices often preceded the carer's sense of urgency, heightened concern, or sheer exhaustion. When carers were vulnerable or distraught and shared their situation with a clinician who recognized their plight, a simple yet brilliant act of compassion offered confidence and hope, renewing carer resolve. Participant examples of this align with Fredrickson's^38^ broaden‐and‐build theory, whereby the positive emotions experienced by the carer in those interactions were novel, even unexpected in thought or activity, building their resources and resolve over time; and Gilligan's^46^ ethic of care where carers' expectations of what was the norm in care were exceeded and the relationship with the giver of that care was strengthened. However, this is not to be confused with a paternalistic approach to healthcare, where the agency and autonomy of a patient or carer are undermined. Rather, it serves to highlight the importance of sensitivity and perceptivity to their needs and preferences to foster empowerment. Brilliant healthcare for children with feeding difficulties and their carers can be realized by considering what exceeds expectations and brings joy and delight to those who deliver and receive healthcare.^34^

The findings demonstrate that brilliance happens, sometimes with the smallest gesture or word. Acts of care that might seem trivial can make a world of difference to others, particularly those who experience stress or adversity—like carers of children who tube‐feed. This is not to suggest that we should expect more from clinicians or carers, many of whom are time‐poor and/or burnout. This offers an opportunity to highlight and reflect on instances of brilliant care that serendipitously occur in the day‐to‐day care of children who tube‐feed. Principally, it is a call to celebrate positive experiences, however small, to acknowledge how people rise above the trials and tribulations often associated with health issues and healthcare, to (re)energise hope and sustain a collective capacity to promote child and carer well being.

### 4.1 Limitations

Despite the value of the findings, three methodological limitations warrant mention. First, the cross‐sectional research design limits the lifespan of the findings. Second, there is no claim that the sample was representative of clinicians or carers who have cared for children with feeding difficulties, within or beyond Australia, particularly given the recruitment strategies and the voluntary nature of participation. And third, social desirability bias might have influenced the findings, whereby the participants altered their contributions to this study to present themselves and/or their situation ‘in a way that is perceived to be socially acceptable, but not wholly reflective of one's reality’.^49^ ^,p.783^ As Nederhof^50^ explained:

When the respondent actually believes a statement to be true of him or herself, even though it is inaccurate, ‘self‐deception’ occurs… On the other hand, a person might purposely misrepresent the truth as a form of impression management motivated by a desire to avoid evaluation.

### 4.2 Implications

Notwithstanding these limitations, the findings have key implications for clinicians, carers, and scholars. For clinicians, the findings highlight strategies to support children with feeding difficulties and their carers—these include listening, being attuned and committed to their well being, sharing decision‐making, sourcing resources, and instilling hope. Incorporating these strategies can capitalize on the interactions with carers without placing additional demands on a clinician's workload. Clinicians should not underestimate the power of a small word or deed as sustenance for carers' resolve the support of their child's health. The findings also point to the importance of normalizing feeding care—this might require education and training, not just for carers, but for anyone with an interest in child well being, including teachers and pastoral care workers. Such efforts are likely to build the skills, knowledge, and confidence required to support children with feeding difficulties and their carers. It is also important to celebrate successes, however small, and commend those who contributed to this success, including the child, their carer, as well as colleagues.

For carers, the findings suggest they should seek out and acknowledge clinicians who listen, involve them in decision‐making processes, and continue to source the resources required (including expertise) to optimize child and carer well being. Given the findings, carers might also benefit from a peer support network. Sharing lived experiences can reduce carer stress, partly because of the opportunity to connect with those who are in the ‘same boat’.^51^ Peer support can normalize experiences that are difficult and associated with stigma, open opportunities to learn practical strategies to manage challenging situations, build capacity, and boost confidence.

For scholars, this article offers fertile ground for research that builds on these findings. Specifically, scholarship is required to clarify the models of care—that is, ‘the way health services are delivered’,^52^ ^,p.3^—that foster brilliant feeding care as well as the conditions required to introduce and sustain these models. These conditions might encompass the leadership styles, the composition of interprofessional teams, the teamwork approaches, and the organizational cultures that enable brilliant feeding care in different contexts, including hospitals, outpatient clinics, and home‐based services, among others. Additionally, given the need to address the negative discourse regarding feeding care, scholarship is required to clarify the methodologies that serve to examine, understand, and promote brilliant healthcare. Given the demonstrated value of participatory methodologies,^53^, ^54^ this might involve the use of video‐reflexive ethnography and/or co‐design approaches with children who tube‐feed, their carers, and the clinicians who work with them.

## AUTHOR CONTRIBUTIONS

Ann Dadich conceived and managed the study, developed the Introduction and Methods and contributed to all sections. Simone Kaplun analysed the data and developed the Results. Cathy Kaplun developed the Discussion. Nick Hopwood and Christopher Elliot reviewed the article.

## CONFLICT OF INTEREST

The authors declare no conflict of interest.

## ETHICS STATEMENT

This study was approved by the Western Sydney University Human Research Ethics Committee (reference number: H13794). All participants provided informed consent.

## Data Availability

Data are not available due to ethical restrictions.

## APPENDIX 1

### Interview schedule for clinicians

1. How did you become interested in feeding difficulties and/or tube‐feeding?
2. What have you found useful when supporting children who tube‐feed and/or their carers, and why?
3. What are your understandings of and experiences with brilliant feeding care?
4. What do you wish you would have known about feeding care, earlier in life, and why?

### Interview schedule for carers

1. What is it like to care for a child who is tube‐fed?
2. What helps or hinders feeding care, and why?
3. What priorities and considerations matter to you, and why?
4. What are your understandings of and experiences with brilliant feeding care?
5. What do you wish you would have known about feeding care, earlier in life, and why?
